# Remote Activation of Host Cell DNA Synthesis in Uninfected Cells Signaled by Infected Cells in Advance of Virus Transmission

**DOI:** 10.1128/JVI.01950-15

**Published:** 2015-08-26

**Authors:** Nora Schmidt, Thomas Hennig, Remigiusz A. Serwa, Magda Marchetti, Peter O'Hare

**Affiliations:** a Section of Virology, St. Mary's Medical School, Imperial College, London, United Kingdom; b Department of Chemistry, Imperial College London, London, United Kingdom; c Department of Technology and Health, Istituto Superiore di Sanità, Rome, Italy

## Abstract

Viruses modulate cellular processes and metabolism in diverse ways, but these are almost universally studied in the infected cell itself. Here, we study spatial organization of DNA synthesis during multiround transmission of herpes simplex virus (HSV) using pulse-labeling with ethynyl nucleotides and cycloaddition of azide fluorophores. We report a hitherto unknown and unexpected outcome of virus-host interaction. Consistent with the current understanding of the single-step growth cycle, HSV suppresses host DNA synthesis and promotes viral DNA synthesis in spatially segregated compartments within the cell. In striking contrast, during progressive rounds of infection initiated at a single cell, we observe that infection induces a clear and pronounced stimulation of cellular DNA replication in remote uninfected cells. This induced DNA synthesis was observed in hundreds of uninfected cells at the extended border, outside the perimeter of the progressing infection. Moreover, using pulse-chase analysis, we show that this activation is maintained, resulting in a propagating wave of host DNA synthesis continually in advance of infection. As the virus reaches and infects these activated cells, host DNA synthesis is then shut off and replaced with virus DNA synthesis. Using nonpropagating viruses or conditioned medium, we demonstrate a paracrine effector of uninfected cell DNA synthesis in remote cells continually in advance of infection. These findings have significant implications, likely with broad applicability, for our understanding of the ways in which virus infection manipulates cell processes not only in the infected cell itself but also now in remote uninfected cells, as well as of mechanisms governing host DNA synthesis.

**IMPORTANCE** We show that during infection initiated by a single particle with progressive cell-cell virus transmission (i.e., the normal situation), HSV induces host DNA synthesis in uninfected cells, mediated by a virus-induced paracrine effector. The field has had no conception that this process occurs, and the work changes our interpretation of virus-host interaction during advancing infection and has implications for understanding controls of host DNA synthesis. Our findings demonstrate the utility of chemical biology techniques in analysis of infection processes, reveal distinct processes when infection is examined in multiround transmission versus single-step growth curves, and reveal a hitherto-unknown process in virus infection, likely relevant for other viruses (and other infectious agents) and for remote signaling of other processes, including transcription and protein synthesis.

## INTRODUCTION

Many viruses inhibit host macromolecular synthesis to suppress cellular antiviral responses or reduce competition from synthesis of host products (1). Viruses also manipulate host autophagic pathways (2), induce and suppress apoptosis (3), and usurp DNA repair pathways (4). The host cell cycle is also modulated by virus infection and can be stimulated or suppressed, depending on the virus (5). Small DNA viruses, including papillomaviruses and adenoviruses, modulate the host G_1_/S-phase transition to stimulate cell cycle-regulated transcription and/or S-phase DNA synthesis and thus support virus genome replication (5–7). On the other hand, large DNA viruses such as the herpesviruses encode their own DNA synthetic apparatus and enzymes for nucleotide production. In the case of herpes simplex virus (HSV), in addition to seven essential replication proteins (8–14), other viral and host proteins localize to segregated replication compartments to promote origin-specific virus DNA replication (see review in reference 15). Moreover, HSV generally suppresses host cell DNA synthesis or blocks the transition from G_1_ to S phase (12) and is thought to interfere with the cell cycle at several distinct phases (16–19; reviewed in reference 20).

All of the events cited above occur within the virus-infected cell itself. Generally, virus manipulation of the intracellular environment is effected either by early events associated with attachment to the host cell, by structural components of the infecting virus, or by *de novo*-expressed virus products. Apart from the well-known processes of paracrine/juxtacrine signaling to uninfected cells in antiviral responses, the prospect that additional modulation of other types of cellular metabolic processes occurs by paracrine signaling to uninfected cells has significant implications for our general understanding of virus-host interactions and the events which impinge on the outcome of infection.

The development of azide- or alkyne-derivatized metabolic precursors combined with cycloaddition to biorthogonal capture reagents is increasingly being exploited in various approaches to biological processes and more recently to mechanisms in infection and immunity (21–25). Here, we use such techniques to study the spatiotemporal distribution of virus and host cell DNA synthesis when infection is initiated in a single cell and followed by progressive transmission to neighboring cells. We show that while HSV disrupts host cell DNA synthesis and promotes virus DNA synthesis in the infected cell itself, it also induces paracrine signaling on control of host cell DNA synthesis in distant uninfected cells, resulting in a propagating wave of DNA synthesis in advance of virus infection. The results are relevant not only for HSV and potentially for viruses in general; they reveal differences in host cell processes when studied in progressive transmission models versus single-step growth cycles and could also indicate that similar events occur in other aspects of cellular metabolism, e.g., transcription and protein synthesis.

## MATERIALS AND METHODS

### Cells and viruses.

Vero cells were grown in Dulbecco's modified minimal essential medium (DMEM; Gibco) containing 10% newborn calf serum (NCS) and penicillin-streptomycin. RPE-1 cells, a human telomerase-immortalized retinal pigment epithelial (RPE) line, were grown in DMEM–F-12 (Sigma) supplemented with 200 mM glutamine, 10% fetal bovine serum (FBS), and penicillin-streptomycin. Rabbit skin cells (RSC) were grown in DMEM containing 10% fetal bovine serum (FBS) and penicillin-streptomycin. Virus strains were HSV-1[17] or HSV-1[17].ICP0-YFP, a derivative of HSV-1[17] which expresses the immediate early protein ICP0 fused to yellow fluorescent protein (YFP) (26). Other strains included HSV-1[KOS] and HSV-1[KOS].VP1-2ΔNLS, which lacks the core nuclear localization signal (NLS) of VP1-2 as previously described (27). Routine plaque assays were performed in the presence of pooled neutralizing human serum (Sigma) at 2% or clinical-grade purified neutralizing human immunoglobulin (IVIg; Carimune NF, nanofiltered, human immune globulin; CSL Behring) at 2 mg/ml, having demonstrated complete neutralization of extracellular virus at this dose (>6-log reduction in virus titer). High-multiplicity infections were performed at a multiplicity of infection (MOI) of 5 or 10. For inhibition of virus DNA synthesis, phosphonoacetic acid (PAA) was used at a final concentration of 400 μg/ml added as indicated in the figure legends.

For analysis of paracrine signaling (see Fig. 7), a dual chamber system (Anapore) was used consisting of an upper insert chamber in which cells (donor cells) were plated on a supporting membrane of defined pore size (0.02 μm), inserted into a lower chamber containing a monolayer of test cells. The upper chamber base membrane, with an 0.02-μm pore size, prevented virus migration through it, and in addition, the cells were plated in neutralizing serum for inhibition of any infectious virus passage through to the bottom chamber. The upper chamber cells were infected at defined multiplicities (Fig. 7) or mock infected, placed in the dual chamber system, and incubated for 20 h. Ethynyl deoxycytidine (EdC) was then added for a standard labeling period, and cells in the lower test chamber were then fixed and processed for analysis of DNA synthesis as described below.

### Immunofluorescence studies.

Immunofluorescence analysis was performed exactly as described previously (28). Samples were collected at times indicated, washed with phosphate-buffered saline (PBS), and fixed either with paraformaldehyde (4%) followed by permeabilization with 0.5% Triton X-100 or by methanol (5 min at −20°C). Samples were blocked with PBS containing 10% NCS or a mix of 5% goat serum, 5% NCS, and 2% bovine serum albumin (BSA), for 1 h at room temperature. Primary antibodies for the antigens were anti-ICP4 antibody (Virusys) used at 1:400 and anti-ICP8 antibody used at 1:400. DNA was stained with 4′,6-diamidino-2-phenylindole (DAPI; Sigma), and coverslips were mounted in Mowiol supplemented with 2.5% 1,4-diazabicyclo(2,2,2)octane (DABCO). Images were taken using an Axiovert 135 TV microscope using Zeiss 10×, 40× LD, or 63× lenses (Plan-Apochromat, 1.4 numerical aperture) and captured using a Retiga 2000R camera with Image Pro Plus software or with a Zeiss laser scanning confocal microscope (Zeiss Pascal).

### Ethynyl nucleotide labeling, click chemistry, and image analysis.

We initially evaluated the effect of EdC or EdU on the growth and doubling of uninfected cells and on HSV replication. There was a modest effect of EdU on both cell growth and virus yield but virtually no effect of EdC on plaque numbers and plaque size in multicycle analysis or virus yield in single-step analysis. We optimized protocols for nucleoside incorporation, click chemistry, and fluorescence detection as follows. Cells on coverslips were mock infected or infected with HSV by normal procedures (MOI, 10) and incubated in DMEM-2% NCS. At the times indicated, EdC was added at a final concentration of 5 μM for a standard labeling time of 4 h unless otherwise stated. The cells were then washed in cold PBS and fixed in 4% paraformaldehyde for 15 min, quenched with glycine (0.3 M) or ammonium chloride (25 mM), and permeabilized in 0.5% Triton X-100 at room temperature for 5 min. For localization of specific antigens, an immunofluorescence assay with primary and secondary antibodies was carried out as described above. The samples were blocked in 10% calf serum and then subjected to click reaction in a buffer prepared freshly in each case (premixed for 2 min) and containing 10 μM Alexa Fluor 488-azide (Sigma-Aldrich), 1 mM CuSO_4_, 10 mM sodium ascorbate, 10 mM aminoguanidine, and 1 mM tris(3-hydroxypropyltriazolylmethyl)amine (Sigma-Aldrich) in PBS, pH 7.4. The reaction was then allowed to proceed by incubation at room temperature in the dark for 90 to 120 min. After washing in PBS, the cells were counterstained with DAPI (Sigma) and coverslips were mounted in Mowiol supplemented with 2.5% DABCO. Images were taken as described above. For quantitation, total cell numbers were enumerated from the nuclear DAPI staining in one channel and EdC-positive cells were enumerated from nuclear staining (above background set in the absence of EdC). Images were quantitated manually (for total numbers and EdC-positive numbers) from the images or, for more quantitative assessment, using the thresholding and segmentation modules of Image Pro Plus. In this workflow, areas of individual objects (nuclei) are defined from the DAPI staining and then total intensity above background is reported in the corresponding object area for EdC incorporation. For the data in Fig. 7, the range of counts for EdC intensity from minimum to maximum was binned and the numbers of cells in each bin were then returned. For these analyses, numerous independent fields and hundreds or thousands of cells were quantified.

In this work, the majority of figures are illustrated using low-magnification microscopy for ease of inspection of numerous cells in a field and to discern the main points of the work, which are visible only at low magnification. Certain data are illustrated at higher magnifications for qualitative points concerning intracellular distribution of DNA and protein components.

## RESULTS

### DNA synthesis analyzed by ethylene nucleoside incorporation in uninfected and HSV-infected cells.

**FIG 1:**
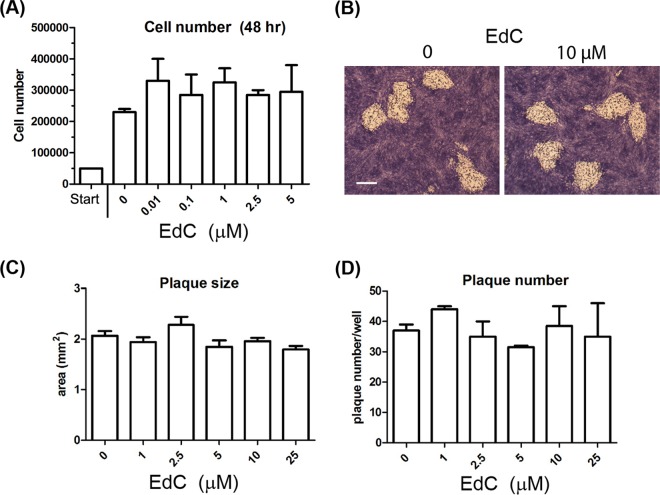
Lack of effect of EdC on cell growth and virus replication. (A) RPE cells (approximately 5 × 10^4^) were counted and plated in duplicate in normal growth medium containing 10% NCS. After 24 h, medium without or with increasing concentrations of EdC was added as indicated. After a further 48 h, cells were harvested, and viable cell numbers were evaluated using trypan blue exclusion EdC labeling for spatial analysis of uninfected and infected cell DNA synthesis. (B) RPE cells were infected with HSV at a low MOI with approximately 40 PFU per well of a 12-well cluster plate. After 2 to 3 h, the medium was removed and replaced with medium containing increasing doses of EdC as indicated, and infected cultures were incubated in the continued presence of the label. After 48 h, cells were fixed, and plaque number and size were evaluated. A typical individual plaque is shown in panel B, with average plaque area and number indicated in panels C and D, respectively.

Analysis of DNA synthesis by labeling with alkyne-derivatized nucleotides and cycloaddition to azide-coupled fluorochromes has been evaluated in several systems (23, 29, 30). In agreement with previous work (23, 31), EdC (ethynyl deoxycytidine) was at least as sensitive for detection as, if not more than, EdU (ethynyl deoxyuridine) (data not shown). We also found no significant effect of EdC on cell replication at doses required for detection of DNA synthesis (Fig. 1A), no effect on virus yields from single-step growth curves, nor any effect even over 2 to 3 days on virus plaque numbers or size (Fig. 1B to D). Further experiments were therefore performed with EdC labeling. The majority of experiments were performed in human retinal pigment epithelial (RPE) cells unless otherwise stated. Experiments confirming observations (see below) were also performed in Vero cells and rabbit skin cells (RSC).

**FIG 2:**
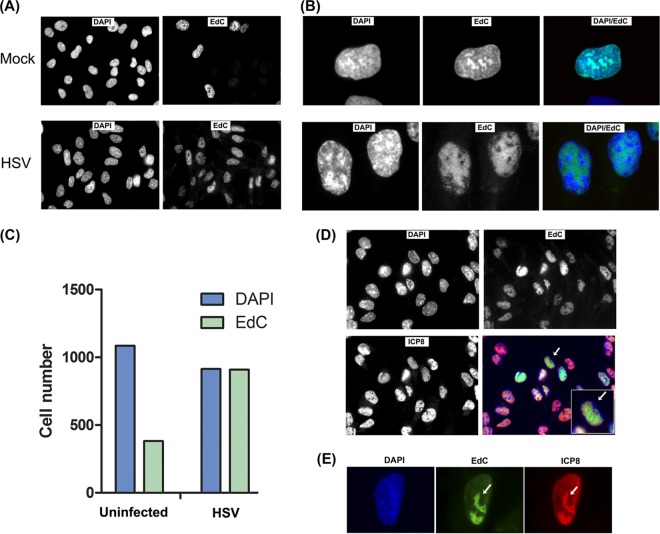
EdC labeling of host and virus DNA synthesis in uninfected and infected cells. (A) Typical fields showing incorporation of EdC from 4 to 8 h after mock infection or HSV infection (MOI of 5) in RPE cells. Cells were counterstained with DAPI. (B) Higher-magnification image (63× objective) showing qualitative features of the localization of EdC incorporation in uninfected or infected cells compared to total DNA. (C) Quantitative analysis of EdC incorporation in mock-infected or HSV-infected cells as a percentage of total cell count. (D) A typical field of HSV-infected cells simultaneously analyzed for total cell DNA (DAPI), EdC incorporation, and ICP8 localization. The inset shows a higher magnification of a cell showing typical features of bulk DNA margination, ICP8 distribution, and EdC incorporation as discussed in the text. (E) A single confocal section taken with a 63× objective and zoom 4 showing EdC incorporation into HSV replication compartments and colocalization with ICP8 as discussed in the text.

EdC labeling (5 μM) of asynchronously growing RPE cells for 4 h allowed ready detection of DNA synthesis in approximately 25 to 30% of cells (Fig. 2A, Mock). Qualitatively, the pattern of EdC incorporation was similar to that previously reported (23, 29), showing one of a few patterns proposed to reflect the approximate stage within S phase (Fig. 2A and B). An example showing total DNA staining (DAPI) and EdC incorporation is shown in Fig. 2B. In contrast, in HSV-infected cells (multiplicity of infection, 10), virtually every cell was positive for EdC incorporation by 8 h after infection (Fig. 2A, HSV). The percentage of EdC-positive cells in several hundred cells is summarized in Fig. 2C, showing ongoing DNA synthesis in virtually every infected cell, compared to the approximately 30% observed in uninfected cells. Moreover, the pattern of incorporation in infected cells was qualitatively distinct from that seen in uninfected cells. An example is shown in Fig. 2D, showing total cell nuclei (DAPI), ICP8 localization, and EdC incorporation in the same field. The results show the previously documented features of HSV DNA replication compartments (12, 32, 33), including EdC colocalization with the DNA replication protein ICP8 and exclusion from DAPI-dense areas of cellular DNA and areas adjacent to the nuclear rim where cellular DNA is marginated (Fig. 2B and D, inset cell marked with arrow). These features are also clearly observed in the higher-magnification image (Fig. 2E).

### EdC incorporation into replication compartments after single-particle infection.

**FIG 3:**
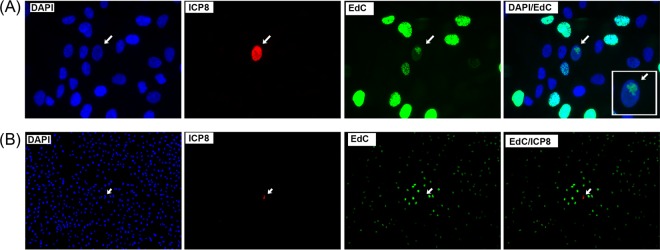
EdC labeling of virus DNA replication compartments initiated after infection in a single cell. (A) Cells were infected at an extremely low MOI, approximately 1/4,000 cells infected; labeled with EdC from 4 to 8 h; and processed for total cell number and DNA (DAPI), ICP8 expression, and EdC incorporation. A single initially infected ICP8+ve cell is detected (arrow) surrounded by numerous uninfected cells. In this cell, a single focus of EdC is observed. (B) A field now imaged at low magnification (10× objective) in order to show a single infected cell (ICP8+ve) in a field of approximately 700 to 800 cells. The single ICP8+ve cell is immediately surrounded by cells showing elevated levels of EdC incorporation as discussed in the text.

We next examined whether virus DNA synthesis could be detected after infection with a single initiating particle, i.e., at an extremely low multiplicity of infection where only one initially infected cell would be expected in several thousand cells. The results demonstrate that early after such infection (within 6 to 8 h), individual infected cells could be identified by the presence of ICP8 (Fig. 3A, ICP8, arrow), surrounded by uninfected cells. The localization of EdC in such single infected cells was qualitatively distinct. A single focus of EdC incorporation within the nucleus was seen, usually at the periphery, combined with substantially reduced incorporation in the remainder of the nucleus (Fig. 3A, EdC, arrow, and DAPI/EdC, inset; see also single-focus inset in Fig. 7). Such a pattern was observed only in ICP8-positive (ICP8+ve) cells and, consistent with previous data from high-multiplicity experiments (12), represents a progressing virus DNA replication compartment, in this case emanating from a single genome beginning to replicate. The general reduction in EdC incorporation, outside the focus of the developing virus replication compartment itself, is consistent with previous data showing that HSV infection blocks cellular G_1_-to-S-phase progression in infected cells (12). However, while our initial intention was to study spatial aspects of DNA synthesis in cells that had been initially infected by only a single particle, observations made during the course of these experiments directed the focus of our investigation to other processes, which are the subject of the remainder of this work.

### HSV infection induces a zone of elevated cellular DNA synthesis in uninfected cells.

**FIG 4:**
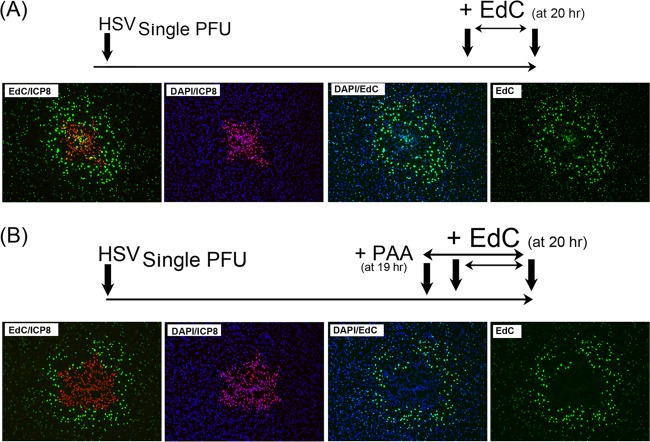
HSV infection induces a zone of elevated host cell DNA synthesis in uninfected cells. (A) Cells were infected at an extremely low MOI, approximately 1/4,000 cells infected; labeled with EdC from 20 to 24 h; and processed for total cell number and DNA (DAPI), ICP8 expression, and EdC incorporation. The panels show different combinations of channels as labeled for ease of inspection of the results. (B) As for panel A but with the prior addition of the virus DNA synthesis inhibitor PAA (400 μg/ml), 1 h before addition of EdC.

During these experiments, we also discovered a distinct feature in the spatial organization of DNA synthesis. Thus, significantly higher levels of DNA synthesis were routinely observed in uninfected cells surrounding the single initially infected ICP8+ve cells than were observed in more distant cells or in uninfected cells (more clearly seen in the lower-magnification view of a typical experiment [Fig. 3B, ICP8, arrow]). This gradient of DNA synthesis could be seen as early as 8 h after single-particle infections. We repeated these experiments, pulse-labeling with EdC at a later time (20 to 24 h) when infection progressed to numerous cells (Fig. 4A). The results show a pronounced zone of cells, surrounding the ICP8+ve infected cell boundary, in which elevated levels of DNA synthesis were observed with a gradient extending to distant cells. While levels of ongoing DNA synthesis were significantly increased in this zone, the percentage of cells synthesizing DNA remained similar to or only marginally increased over that seen in uninfected cell monolayers. This result was obtained independently of the virus antigen used to detect the central infected cells and in several cell types, including RSC and Vero cells (see Fig. S1A in the supplemental material).

**FIG 5:**
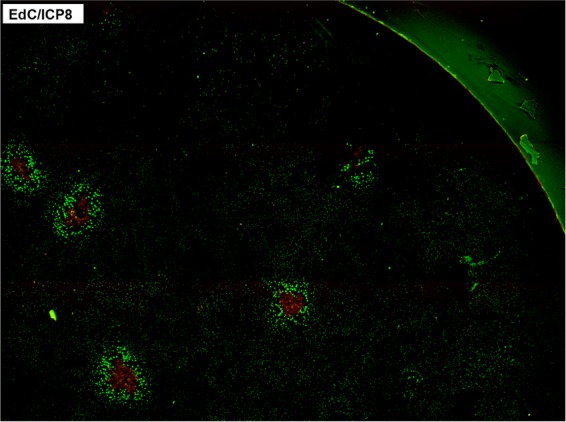
HSV induction of elevated host cell DNA synthesis in progressing plaques. RPE cells were infected as described for Fig. 4, labeled with EdC from 20 to 24 h, and processed for ICP8 expression and EdC incorporation. Multiple images were captured on a motorized stage and tiled together into one image covering a quarter of the entire dish and showing 5 developing foci of virus spread, all exhibiting elevated DNA synthesis in the extended boundary beyond virus infection.

To confirm that the increased EdC incorporation represented cellular DNA synthesis, we repeated these experiments, adding the virus-specific DNA synthesis inhibitor phosphonoacetic acid (PAA) prior to the EdC pulse (Fig. 4B). PAA had no significant effect on the induction of DNA synthesis in the numerous cells surrounding the infected cell boundary while suppressing DNA synthesis in the central ICP8+ve cells (Fig. 4B, DAPI/EdC merged channel and also EdC single channel). The intracellular patterns of elevated DNA synthesis (Fig. 3 and 4) exhibited a spatial distribution of DNA synthesis similar to that normally seen in uninfected cells, only at higher overall levels. This result was typical of all developing plaques, as illustrated by a single tiled image showing a significant portion of the entire monolayer with five developing plaques (Fig. 5), and was also observed during infection with HSV-2 (see Fig. S1B in the supplemental material).

### HSV induces a propagating zone of elevated cellular DNA synthesis.

**FIG 6:**
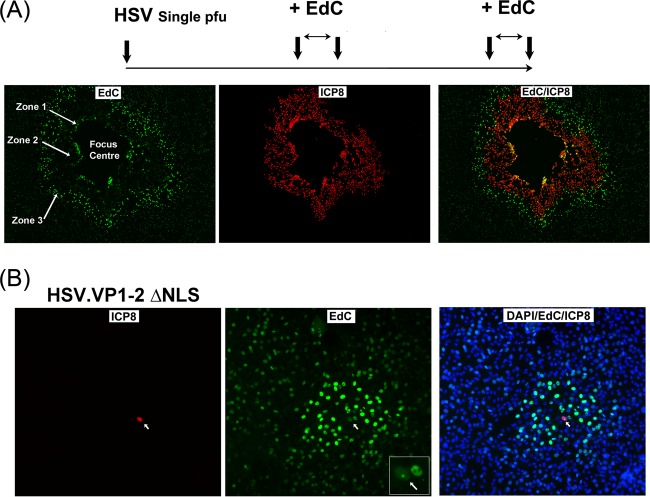
HSV infection induces a propagating wave of elevated DNA synthesis in uninfected cells. (A) Cells were infected at low MOIs, pulsed with EdC at 20 h, chased in the absence of EdC, and then pulsed again at 40 h. The progressing infection can be assigned to different zones of activity as discussed in the text. (B) Cells were infected at low MOIs with the mutant HSV-1.VP1-2ΔNLS, pulse-labeled with EdC from 20 to 24 h postinfection, and processed. A single infected ICP8+ve cell (arrow) is detected, within which is a single focus of viral EdC incorporation (see inset, cell marked with arrow, EdC panel). This cell was surrounded by numerous uninfected cells exhibiting elevated DNA synthesis.

The induction of cellular DNA synthesis appeared as an early event in response to even a single infected cell. We wished to address the duration of this mechanism using a combined pulse-chase-pulse regime (Fig. 6A). After single-particle infection, cells were pulsed with EdC at 20 h, washed and incubated for a further 20 h to allow virus progression, and then pulsed again with EdC and analyzed. We anticipated that if induction of DNA synthesis was maintained, we should see a second external zone of elevated DNA synthesis surrounding the infected cells. As shown in Fig. 6A, this is exactly what was observed. (The various zones defined below are labeled only on the EdC panel for ease of inspection.) Over the course of 44 h, the center of the plaque showed more extensive cytopathic effect (focus center) and was lost during processing, but the remaining cells from the first zone of elevated DNA synthesis (zone 1) could be seen, now with infected cells (ICP8+ve) extending beyond. Some of the remaining cells in this initial zone 1 could be seen labeled for both cell DNA synthesis and ICP8 (EdC/ICP8 merged panel). Extending beyond this (zone 2) are the infected cells now chased in the absence of EdC, i.e., ICP8+ve but with little EdC. The cells at the periphery of zone 2 show weak EdC labeling representing viral DNA synthesis during the second pulse-labeling period. However, at the extended border beyond zone 2 (beyond the zone of infection marked by ICP8), we now again see elevated DNA synthesis in numerous uninfected cells (zone 3), with a gradient extending to the more distant uninfected cells. Thus, at least for up to 2 days, during multiple rounds of infection there is a continuous wave of induction of cellular DNA synthesis in uninfected cells in advance of the progressing infection.

### A nonreplicating virus induces elevated cellular DNA synthesis in uninfected cells.

We could detect elevated cellular DNA synthesis early after a single-particle infection in the absence of any significant virus protein synthesis in the surrounding cells and in the presence of PAA outside plaque boundaries. These results indicated that stimulation of DNA synthesis required neither infection nor virus gene expression in activated cells. Nevertheless, it remained possible, though unlikely, that events associated with infection could be involved. To rule this out, we examined single-particle infection with a virus incapable of spreading to surrounding cells (27). This virus, HSV-1.VP1-2ΔNLS, when produced on complementing cells, can infect a cell and make particles, but these particles do not productively infect surrounding cells due to a defect in capsid transport (27). Typical results are shown in Fig. 6B, where a single mutant virus-infected cell is detected expressing ICP8. In this infected cell, the single initiating virus replication compartment can be observed (Fig. 6B, EdC, arrow and inset). This cell is surrounded by cells exhibiting elevated DNA synthesis, with a gradient extending to more distant cells with no virus gene expression and which will not be infected. Since this mutant produces particles, albeit ones unable to productively infect cells, we repeated this regime with another disabled virus, HSV-1ΔVP1-2.VP26.GFP, in which the VP1-2 gene is deleted and which does not assemble or release infectious particles (34), with similar results (see Fig. S1C in the supplemental material). Together, these data provide strong evidence that the stimulation of DNA synthesis results from a paracrine effect, signaled by infected cells to surrounding uninfected cells.

### Paracrine signaling from infected cells stimulates cellular DNA synthesis.

**FIG 7:**
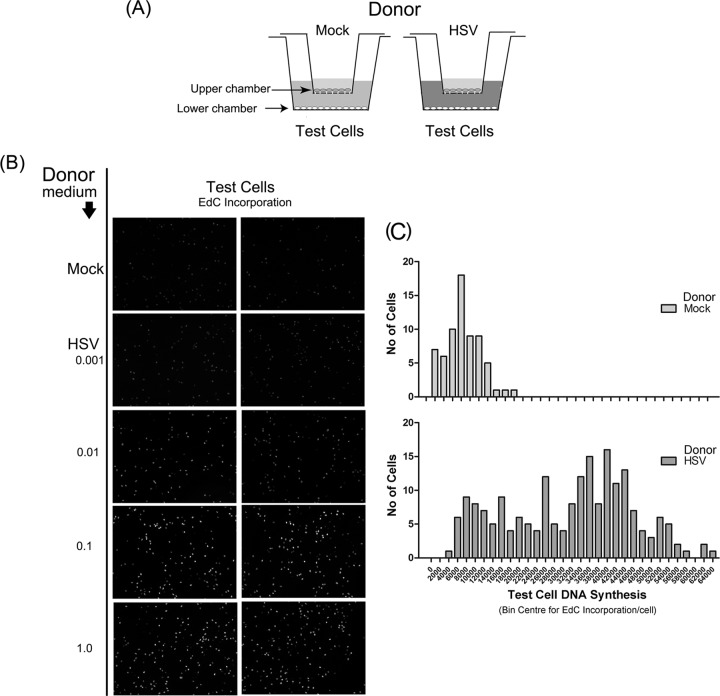
HSV-infected cells induce a paracrine mediator of elevated cellular DNA synthesis. (A) Diagram of a dual chamber system to examine paracrine stimulation of DNA synthesis. (B) The donor cell chamber was mock infected or infected with HSV at different MOIs as indicated, and 20 h later, the donor chamber was removed and test cells were pulse-labeled with EdC for 4 h. Two individual fields (low-magnification, 10× objective) are illustrated for each condition. (C) Quantitative analysis of EdC incorporation in test cells in medium from mock-infected or HSV-infected donor cells performed as described in Materials and Methods.

To next pursue the possibility of a paracrine mechanism initiating from the infected cell, we used an insert culture system in which the infected effector cells were not in contact with the test cells. Cells grown in an insert containing a membrane with 20-nm pores were infected or mock infected (i.e., donor cells) and incubated with the recipient test cells in the lower chamber (Fig. 7A). Approximately 20 h after infection, the inserts were removed and the test cells were pulse-labeled with EdC. The results (Fig. 7B) demonstrate a significant increase in test cell DNA synthesis when exposed to HSV-infected donor medium compared to uninfected cell donor medium. This increase also showed a dose response in relation to the numbers of initially infected cells in the donor cell population (cf. test cell DNA synthesis levels when donor cells were infected at MOIs of 0.001, 0.01, and 0.1). The results were quantified and expressed in numerous test cells, binned into ranges, and plotted for mock-infected or HSV-infected donor cells (Fig. 7C). The results show a substantial increase in EdC incorporation in test cells exposed to donor medium from infected cells. This result cannot be explained by virus infection *per se* in the lower test chamber. First, infection would yield a focus of increased DNA synthesis emanating from an infected cell. This was not observed. Second, HSV will not pass through a 20-nm-pore membrane. Third, the cultures were incubated in the presence of neutralizing antibody. Finally, no virus-infected cells were detected in the test monolayer. Taken all together, our results indicate that, for induction of host DNA synthesis during progressive rounds of infection, the activated cells do not need to be in contact with infected cells and that a paracrine mechanism operates whereby signal(s), even from a single infected cell, promotes elevated DNA synthesis in surrounding uninfected cells.

## DISCUSSION

The results of this work have several implications, specifically for processes involved in HSV replication and generally for consideration of mechanisms involved in virus replication. Such processes are frequently studied and deduced from single-step growth analysis and, based on this work, may be qualitatively distinct when studied during progressive rounds of transmission where the environment of a susceptible uninfected cell is modified by exposure to infected cells.

Previous work from high-multiplicity analysis convincingly shows that HSV actively blocks various stages of the cell cycle, including G_1_-S transition and mitosis, although if cells are infected during active S phase, continued DNA synthesis may not be blocked (12, 17). It has been concluded that HSV infection is independent of the cell cycle, and if anything, infection suppresses cell cycle progression, presumably to promote an optimal environment for virus replication. In the context of the infected cell itself, our results are in agreement with these observations. Nevertheless, what has not previously been demonstrated or appreciated are the results shown here of paracrine stimulation mediated by infected cells to surrounding uninfected cells. Two questions present themselves, first on the implication for the outcome of virus infection and second on the possible mechanisms involved.

With regard to the first question, the most reasonable proposition would be that activation of host DNA synthesis might promote virus infection by any of a number of not mutually exclusive mechanisms, including production of cellular replication components or establishing particular intranuclear niches. We have attempted to show, e.g., by increased plaque numbers or faster spread, that conditioned medium promotes more efficient infection but have not observed a significant increase in these parameters. It is possible that such an outcome may be demonstrated only under suboptimal conditions, e.g., with debilitated or poorly replicating viral mutants, where the efficiency of initiation of infection or the kinetics of spread may reveal an augmented outcome. It is also possible that any effect may not be readily demonstrable in the otherwise very permissive system of tissue culture infection by HSV. It may be that the physiological relevance of this paracrine effect is reflected more in the situation of initial infection *in vivo* or of reactivation in neuronal cells which are not normally transiting through S phase or surrounding supporting cells in the three-dimensional environment. Ultimately, investigation of the influence on the outcome of infection will require knowledge of the virus and/or host components involved in order to interfere with the pathway and explore the consequences. We note also that notwithstanding HSV repression of S-phase progression, many lines of evidence indicate a positive association between the cell cycle/cellular DNA synthesis and the outcome of herpesvirus replication. For example, cell cycle-dependent kinases are known to be required for replication of several herpesviruses, including HSV and human cytomegalovirus (HCMV) (35, 36); HCMV manipulates quiescent cells, producing a pseudo-S-phase environment to support viral replication (37, 38); and certain viral proteins from Epstein-Barr virus (EBV) can actively stimulate E2F-responsive transcription and S-phase progression (39).

With regard to the complex question of mechanism, while direct cell-cell communication remains possible, our results indicate a paracrine effect on cellular DNA synthesis produced from infected cells. Such an effect could be due to a virus- or host cell-encoded product which activates host DNA synthesis. Whether this is a conventional type of growth factor and whether it is secreted as an individual component or, e.g., as some form of vesicle such as an exosome await further mechanistic and biochemical studies. In terms of the processes within an infected cell itself to elicit the response in uninfected cells, preliminary data from assays in which single-particle infection was carried out in the presence of PAA, added from the time of infection, did not show infection to result in zones of elevated DNA synthesis surrounding the single infected cell. This indicates that infection *per se* is insufficient and that late events or accumulating early events are required.

Interestingly, it has been shown in models of ocular pathogenesis that HSV induces the secretion of cytokines, including, e.g., vascular endothelial growth factor (VEGF), which are implicated in blood vessel migration but also capable of promoting growth in nonendothelial cells (40). It is possible that these processes are linked and that the stimulation in DNA synthesis observed here is integrated with other responses *in vivo*. Nonetheless, by quantitatively evaluating the percentage of S-phase cells in the zones exhibiting increased synthesis compared to total cell numbers in the zones (data not shown), we find little alteration from the percentage of S-phase cells in the uninfected cell population. The results may indicate that the process is not one advancing the G_1_-to-S onset or duration but rather one influencing the overall abundance of DNA synthesis in the cell. Such a mechanism might involve an increase in the efficiency of origin firing or in the suppression of certain cell cycle controls limiting DNA replication. It is noteworthy that adenovirus infection, which stimulates S-phase DNA synthesis, has been suggested to involve uncontrolled firing of DNA replication origins by suppressing cyclin A and geminin, which otherwise suppress genome rereplication (5, 41). It is possible, therefore, that stimulation represents a perturbation of cell control mechanisms, e.g., in timing of origin firing or rereplication, rather than a stimulation of normal DNA synthesis. Other processes, including, e.g., DNA damage/repair, could also be involved.

While a full mechanistic understanding awaits further analysis, these results on the process itself are immediately relevant for HSV, for herpesvirus biology, and potentially for other classes of viruses. They also reveal distinct differences in host cell processes studied in progressive transmission models versus single-step growth cycles and could also indicate that similar events take place in other aspects of cellular metabolism, including, for example, transcription and protein synthesis. We propose, therefore, that these observations advance our fundamental concepts about processes involved in perturbation of host processes during virus infection. They are likely to be highly relevant in the broader virology context and, while raising many questions, open new lines of investigation on the diverse pathways by which viruses manipulate host processes to influence infection outcome and disease progression.

## Supplementary Material

Supplemental material
