# Supplementary material for: Remote Activation of Host Cell DNA Synthesis in Uninfected Cells Signaled by Infected Cells in Advance of Virus Transmission
Source: J Virol. 2015 Aug 26;89(21):11107–15. doi: 10.1128/JVI.01950-15 (PMC4621119; doi:10.1128/JVI.01950-15)
Supplement: Supplemental material [file supp_89_21_11107__index.html]

Supplemental material 

# Remote activation of host cell DNA synthesis in uninfected cells signalled by infected cells in advance of virus transmission

## Supplemental material

- Supplemental file 1 -

  Fig. S1 (HSV infection induces elevated DNA synthesis in multiple cell types.)

  PDF, 116K
