# Supplementary material for: Remote Activation of Host Cell DNA Synthesis in Uninfected Cells Signaled by Infected Cells in Advance of Virus Transmission
Source: J Virol. 2015 Aug 26;89(21):11107–15. doi: 10.1128/JVI.01950-15 (PMC4621119; doi:10.1128/JVI.01950-15)
Supplement: Supplemental material [file JVI.01950-15_zjv999090935so1.pdf]

Supplementary Information

Figure S1

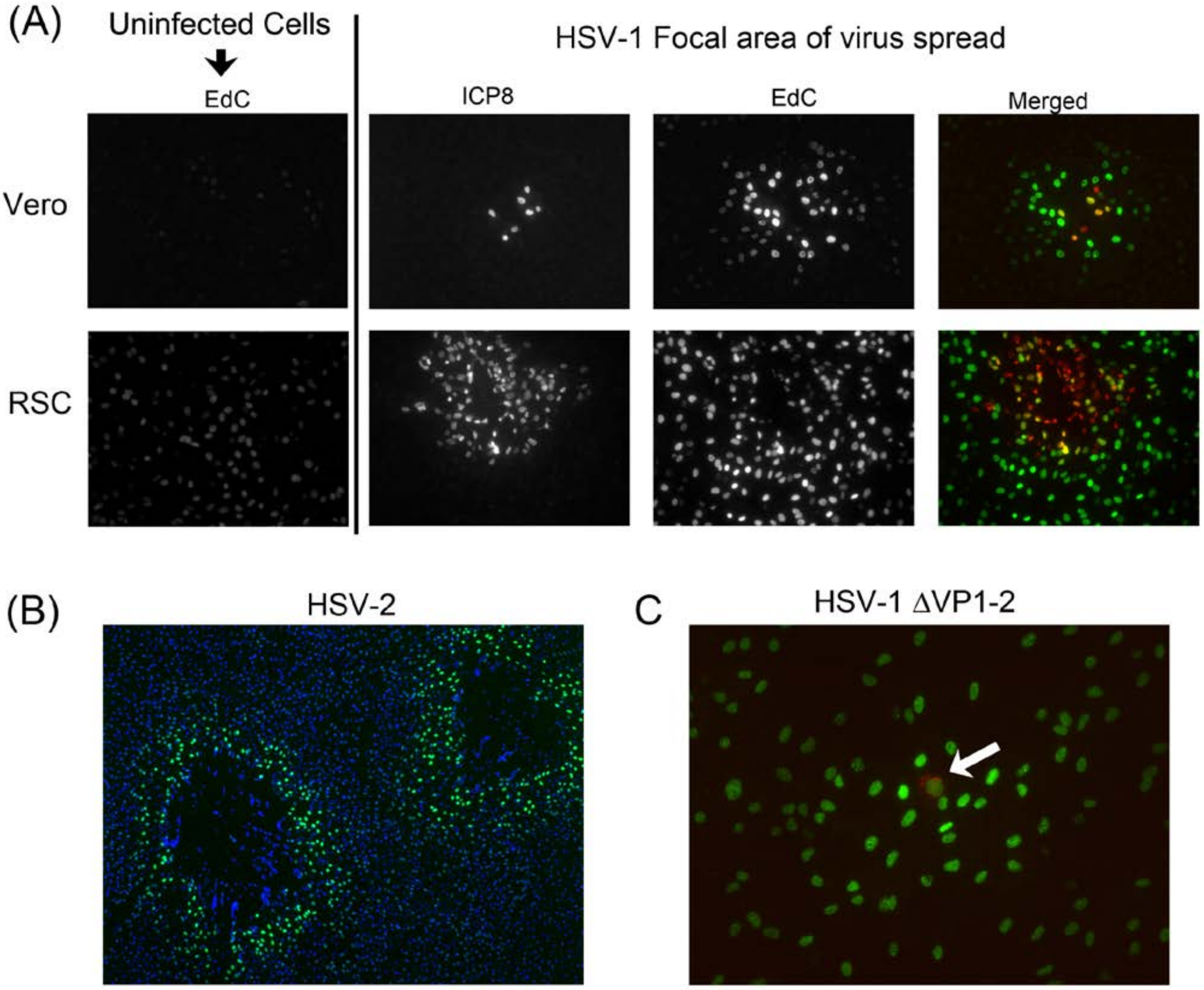

Figure S1. HSV infection induces elevated DNA synthesis in multiple cell types uninfected cells.

(A) Vero cells or RSC cells were infected with HSV-1 at low moi, pulsed with EdC from 20-24 hrs and processed for ICP8 expression and EdC incorporation, shown in grey scale and merged in colour (ICP8, red channel; EdC green channel). Representative uninfected areas distant from the focus of infection are illustrated in the left hand panels. (B) Cells were infected with HSV-2 [186] and pulsed with EdC from 20-24 hrs. In this case the cells were not stained for ICP8 expression. HSV-2 plaques generally progressed quicker than HSV-1, and late incorporation of EdC into infected cells within the plaque was at a lower level. However clear, pronounced elevated EdC incorporation in ring zones surrounding the central area of cpe was obvious. (C) Cells were infected at low moi with the mutant HSV-1ΔVP1-2, pulsed labelled with EdC from 20-24 hrs post-infection and processed. A single infected

ICP8+ve cell is detected (red channel, arrowed), surrounded by numerous uninfected cells exhibiting elevated DNA synthesis in a gradient to more distant cells (green channel).
